# Diagnostic utility of zinc protoporphyrin to detect iron deficiency in Kenyan preschool children: a community-based survey

**DOI:** 10.1186/s12878-017-0082-z

**Published:** 2017-07-27

**Authors:** Emily M. Teshome, Andrew M. Prentice, Ayşe Y. Demir, Pauline E.A. Andang’o, Hans Verhoef

**Affiliations:** 1 MRCG Keneba at MRC Unit The Gambia, PO Box 273, Banjul, The Gambia; 2 MRC International Nutrition Group, Faculty of Epidemiology and Population Heath, London School of Hygiene and Tropical Medicine, Keppel Street, London, WC1E7HT UK; 3 Meander Medical Centre, Laboratory for Clinical Chemistry, Maatweg 3, 3813 TZ, Amersfoort, Netherlands; 4 School of Public Health and Community Development, Maseno University, Private Bag, Maseno, Kenya; 5 Division of Human Nutrition and Cell Biology and Immunology Group, Wageningen University, P.O. Box 17, 6700 AA Wageningen, The Netherlands

**Keywords:** Erythrocyte protoporphyrin, Inflammation, Iron deficiency, Kenya, Malaria, *Plasmodium*, Child, Preschool, Zinc protoporphyrin

## Abstract

**Background:**

Zinc protoporphyrin (ZPP) has been used to screen and manage iron deficiency in individual children, but it has also been recommended to assess population iron status. The diagnostic utility of ZPP used in combination with haemoglobin concentration has not been evaluated in pre-school children. We aimed to a) identify factors associated with ZPP in children aged 12–36 months; b) assess the diagnostic performance and utility of ZPP, either alone or in combination with haemoglobin, to detect iron deficiency.

**Methods:**

We used baseline data from 338 Kenyan children enrolled in a community-based randomised trial. To identify factors related to ZZP measured in whole blood or erythrocytes, we used bivariate and multiple linear regression analysis. To assess diagnostic performance, we excluded children with elevated plasma concentrations of C-reactive protein or *α*_1_-acid glycoprotein, and with *Plasmodium* infection, and we analysed receiver operating characteristics (ROC) curves, with iron deficiency defined as plasma ferritin concentration < 12 μg/L. We also developed models to assess the diagnostic utility of ZPP and haemoglobin concentration when used to screen for iron deficiency.

**Results:**

Whole blood ZPP and erythrocyte ZPP were independently associated with haemoglobin concentration, *Plasmodium* infection and plasma concentrations of soluble transferrin receptor, ferritin, and C-reactive protein. In children without inflammation or *Plasmodium* infection, the prevalence of true iron deficiency was 32.1%, compared to prevalence of 97.5% and 95.1% when assessed by whole blood ZPP and erythrocyte ZPP with conventional cut-off points (70 μmol/mol and 40 μmol/mol haem, respectively). Addition of whole blood ZPP or erythrocyte ZPP to haemoglobin concentration increased the area-under-the-ROC-curve (84.0%, *p* = 0.003, and 84.2%, *p* = 0.001, respectively, versus 62.7%). A diagnostic rule (0.038689 [haemoglobin concentration, g/L] + 0.00694 [whole blood ZPP, μmol/mol haem] >5.93120) correctly ruled out iron deficiency in 37.4%–53.7% of children screened, depending on the true prevalence, with both specificity and negative predictive value ≥90%.

**Conclusions:**

In young children, whole blood ZPP and erythrocyte ZPP have added diagnostic value in detecting iron deficiency compared to haemoglobin concentration alone. A single diagnostic score based on haemoglobin concentration and whole blood ZPP can rule out iron deficiency in a substantial proportion of children screened.

**Trial registration:**

ClinicalTrials.gov NCT02073149 (25 February 2014).

**Electronic supplementary material:**

The online version of this article (doi:10.1186/s12878-017-0082-z) contains supplementary material, which is available to authorized users.

## Background

Zinc protoporphyrin (ZPP) is formed in erythrocytes when the iron supply for erythropoiesis is less than required, or when iron utilization is impaired. In such conditions of iron-deficient erythropoiesis, protoporphyrin IX, the immediate precursor of haem, incorporates an atom of zinc rather than iron, resulting in the formation of ZPP instead of haem. Thus, depleted iron stores or a decrease in circulating iron in the bone marrow lead to elevated ZPP concentrations in whole blood or erythrocytes [1, 2].

ZPP can be determined rapidly and at low assay cost by haematofluorometer. It has been used to screen and manage iron deficiency in individual children, but it has also been a recommended marker to assess population iron status in cross-sectional studies, together with haemoglobin concentration [1, 3].

One possible application concerns the use of a single score that captures the diagnostic information of both whole blood ZPP and haemoglobin concentration in a screen-and-treat approach to manage iron deficiency in preschool children. Such diagnostic utility has not been assessed in pre-school children. We found earlier, however, that both whole blood ZPP and erythrocyte ZPP have little diagnostic utility as a screening marker to manage iron deficiency in pregnant women, whether used as single tests or combined with haemoglobin concentration [4].

The present study aimed to identify factors associated with ZPP measured in whole blood or erythrocytes from preschool children. We also assessed the diagnostic performance and utility of ZPP, either alone or in combination with haemoglobin concentration, in detecting iron deficiency defined as plasma ferritin concentration < 12 μg/L in children without inflammation or *Plasmodium* infection.

## Methods

### Study setting and population

The present study made use of samples that were collected at baseline in a randomised placebo-controlled trial to show non-inferiority of home fortification with 3 mg iron as NaFeEDTA compared with 12.5 mg iron as encapsulated ferrous fumarate. The main results of this trial will be reported elsewhere [5]. The study was conducted in children aged 12–36 months from January–December 2014 in Kisumu-West District, Kenya, an area that is located at around 1350 m above sea level. To recruit the children, community health workers compiled a list of parents with children within the eligible age range in a predefined study area and invited parents to bring all of them for screening to the research clinic, where parents were asked to sign an informed consent form.

### Collection of data and samples

We determined weight and height using Salter Scale (UNICEF, catalogue 0145555, Copenhagen, Denmark) and height/recumbent length boards (UNICEF, catalogue 0114500, Copenhagen, Denmark) within 100 g and 1 mm, respectively. Phlebotomists collected 4 mL venous blood in tubes containing Li-heparin. An aliquot of blood was centrifuged, plasma was transferred to a microtube, centrifuged, and stored immediately in liquid nitrogen (−196 °C). The erythrocyte sediment was washed and centrifuged three times with isotonic phosphate-buffered saline. We assessed haemoglobin concentration (HemoCue 301, Ängelholm, Sweden) in duplicate, and zinc protoporphyrin: haem ratio (AVIV haematofluorometer, model 206D, Lakewood NJ, USA) in whole blood and in erythrocytes, each in triplicate. For quality control of the haematofluorometer, we used erythrocyte controls for low, medium and high ZPP values from the manufacturer (Aviv) and as per manufacturer’s instructions. Measurements were within the acceptable range throughout the study.

ZPP in washed erythrocytes is considered a more valid measure of iron-deficient erythropoiesis when compared to ZPP in whole blood because the washing process removes substances dissolved in plasma such as bilirubin and riboflavin that can fluoresce at a wavelength similar to that of ZPP.

We used two rapid diagnostic tests to detect *Plasmodium* antigenaemia. CareStart G0151 (AccessBio, USA; http://www.accessbio.net/) can detect lactate hydrogenase (pLDH) produced by either *P. falciparum* or *Plasmodium* species other than *P. falciparum* (i.e. *P. ovale, P. malariae* or *P. vivax*). CareStart G0171 can detect histidine-rich protein-2 (HRP2), which is produced exclusively by *P. falciparum*.

Plasma iron indicators (concentrations of ferritin and soluble transferrin receptor), inflammation indicators (concentrations of C-reactive protein and *α*-1-acid glycoprotein) and other nutritional markers (concentrations of albumin and vitamin B_12_) were measured on an Abbott Architect C16000 and i2000 SR analyser at Meander Medical Centre, Amersfoort, The Netherlands, with reagents from and as per instructions of the manufacturer.

### Eligibility criteria

After data and blood samples were collected but before randomisation to intervention, children were given pre-medication (3-day courses of dihydroartemisinin-piperaquine and albendazole; a single dose of praziquantel). Children were eligible for enrolment in the trial and the present study if: aged 12–36 months, resident in the study area; parental consent form signed by both parents; not acutely sick or febrile (axillary temperature ≥ 37.5 °C) at the time of recruitment; absence of reported or suspected major systemic disorder (e.g. HIV infection, sickle cell disease); no use of antiretroviral drugs against HIV, rifampicin, carbamazepine, phenytoin or phenobarbital and no twin sibling. Children were excluded if: haemoglobin concentration < 70 g/L (for ethical reasons, because the trial had a placebo arm); severely wasted (weight-for-height z-score < −3 SD); known allergy to dihydroartemisinin-piperaquine, benzimidazole or praziquantel; parent-reported history of using antihelminthic drugs in the 1-month period before the screening date; not at risk of malaria (e.g. children who received chemoprophylaxis against malaria because of HIV infection or sickle cell disease); they received their first dose of dihydroartemisinin-piperaquine at the research clinic and did not complete the prescribed second and third doses at home.

### Sample size determination

We included all children enrolled in the trial in the present study. Our sample size calculations were based on our primary aim to show non-inferiority of the haemoglobin concentration response to home fortification with 3 mg iron as NaFeEDTA compared with 12.5 mg iron as ferrous fumarate intention. Because this aim is irrelevant to the present study, these calculations are not reported here, although they are available elsewhere [5].

### Statistical analysis

#### Definitions

Anthropometric indices were calculated by comparing measurements with the standards WHO Growth Standards [6]. For whole blood and erythrocyte ZPP, we used a cut-off value of 70 μmol/mol haem (2.7 μg/g haemoglobin), corresponding to the 95% upper limit of the reference values for women and children participating in the US National Health and Nutrition Examination Survey (NHANES) II, from which individuals with anaemia, low transferrin saturation and elevated blood lead concentrations had been excluded [1–3]. For erythrocyte ZPP, we also used a cut-off point of 40 μmol/mol haem, which is based on several small studies comparing iron-deficient and iron-replete individuals [7].

We defined iron deficiency as the absence or near-absence of storage iron, indicated by plasma ferritin concentration < 12 μg/L [8]. Because this definition is recommended by WHO to measure population iron status except where inflammation is prevalent [2], we considered it to be valid only in children without inflammation, *Plasmodium* infection, or HIV infection. In addition, we used the following definitions: anaemia: haemoglobin concentration < 108 g/L (i.e. 110 g/L reference range for children aged 6–59 months, at sea level, minus 2 g/L adjustment for an altitude at 1000–1500 m above sea level) [9]; inflammation: plasma concentrations of C-reactive protein concentration > 5 mg/L [10] and/or *α*_1_-acid glycoprotein concentration > 1 g/L [11]; being stunted or wasted: height-for-age or weight-for-height z-score < −2 SD; [6] *P. falciparum* infection: presence in blood of HRP2 or *P. falciparum*-specific pLDH; any *Plasmodium* infection: presence of HRP2 or pLDH specific to either *P. falciparum* or human *Plasmodium* species other than *P. falciparum*; low vitamin B_12_ status: plasma vitamin B_12_ concentration < 150 pmol/L.

#### Description of the study population

We calculated prevalence values for binary variables, means with corresponding SDs for variables with an approximately normal distribution, and quartiles for continuous variables that were not normally distributed. Because some of the plasma markers used (ferritin, albumin, vitamin B_12_) can act as acute phase reactants we also described these characteristics in children without inflammation or *Plasmodium* infection.

#### Factors associated with ZPP

We explored associations between ZPP and personal characteristics (age, sex), inflammation markers, iron markers, *Plasmodium* infection and other plasma markers (albumin and vitamin B_12_ concentrations). Groups were compared assuming t-distributions of ZPP values that were normalised by log-transformation. Exponentiation of results yielded group differences that were expressed as relative differences.

We inspected scatterplots and used simple linear regression analysis to assess associations between ZPP (log-transformed) and explanatory variables with continuous outcomes. Some explanatory factors were untransformed, with the implicit assumption that ZPP values can increase or decrease exponentially with an absolute increment in the explanatory variable; in other cases, log-transformation of the explanatory factor yielded a better model fit, indicating that ZPP values and explanatory variables change at rates that are proportional to their current values. In such cases, variation in the independent variable was expressed as geometric standard deviation, i.e. a dimensionless, multiplicative factor such that dividing or multiplication of the geometric mean by this ratio indicates a variation that is equivalent to subtraction or addition of one standard deviation on a log-transformed scale [12].

We subsequently used multiple linear regression analyses to identify factors that were independently associated with ZPP. Given a linear association between continuous variables, dichotomisation generally results in loss of statistical precision [13]. Thus we preferred to use continuous variables that were shown to be linearly associated with ZPP in the bivariate analyses. Our analysis started with a full model that included haemoglobin concentration, *Plasmodium* infection, and plasma concentrations of ferritin, soluble transferrin receptor, C-reactive protein, *α*_1_-acid glycoprotein, albumin, vitamin B_12_, sex (binary) and age class (binary). All plasma markers except albumin and vitamin B_12_ were log-transformed. Factors were manually eliminated using a backward elimination process with a removal criterion of *p* > 0.05.

#### Diagnostic performance of ZPP to detect iron deficiency

This part of the analysis was restricted to children without inflammation (i.e. plasma concentrations of C-reactive protein <5 mg/L and/or *α*_1_-acid glycoprotein <1 g/L) and without *Plasmodium* infection. We used logistic discriminant analysis to model the probability of iron deficiency as a function of continuous explanatory variables, either alone or combined.

We used the pROC package [14] within R vs. 3.2.0 (www.r-project.org) to produce and analyse receiver operating characteristics (ROC) curves, with comparison of areas-under-the-curve (AUCs) by DeLong’s test for paired curves. Partial AUCs were computed with a correction to achieve a maximal value of 1.0 and a non-discriminant value of 0.5, whatever the range of specificity or sensitivity values. Confidence intervals of estimates for partial areas-under-the-curve (pAUCs) were computed by stratified bootstrapping with 10,000 replicates.

#### Diagnostic utility of ZPP to estimate prevalence of iron deficiency

First, we assessed the diagnostic performance of ZPP to estimate the prevalence of iron deficiency, using two commonly used cut-off points for ZPP, namely whole blood ZPP >70 μmol/mol haem and erythrocyte ZPP >40 μmol/mol haem (see preceding paragraphs). We used Wilson’s method to calculate confidence intervals around proportions [15, 16]; for sensitivity and specificity, and for the pair of predictive values, we calculated 97.5% univariate CIs. The cross-product of these univariate CIs, considered together, form a joint 95% confidence region for both population parameters [17].

Second, as an example, we used our data to explore the utility of using the combination of haemoglobin concentration and ZPP to screen for iron deficiency (Additional file 1), with cut-points chosen to ensure a high sensitivity so that most cases are detected, at the cost of false positives that could be eliminated by further diagnostic tests. Children can be excluded from further testing if negative test results correctly identify children without iron deficiency in the vast majority of cases. Such a strategy may be desirable in community-based surveys with relatively low prevalence of iron deficiency, but also in medical practice with higher prevalence values (because of self-selection). Thus we estimated the proportion of children who could be eliminated from further testing in settings with a prevalence range for iron deficiency of 0%–50%, which probably covers the vast majority of community settings, with arbitrarily selected sensitivity values and negative predictive values of >90%. Because of its ease of measurement, we limited this assessment with ZPP being measured in whole blood.

## Results

We enrolled 338 children into the study.

### Description of the study population

The prevalence of iron deficiency, measured in children without inflammation or *Plasmodium* infection, was 32.1%; when measured in all children (Table 1), this value was 17.1%. Whole blood ZPP > 70 μmol/mol haem occurred in 97.9% and erythrocyte ZPP > 40 μmol/mol occurred in 96.7% of children. The prevalence of anaemia was 55.6%, but there were virtually no children with low vitamin B_12_ status. Inflammation, assessed by plasma concentrations of C-reactive protein and α_1_-acid glycoprotein, occurred in 66.9% of children, and was mostly mild. Of 226 children with inflammation, 98 (43.4%) had elevated concentrations of α_1_-acid glycoprotein with normal C-reactive protein concentrations, 11 (4.9%) had elevated C-reactive protein concentrations with normal concentrations of α_1_-acid glycoprotein, and 117 (51.8%) had elevated concentrations of both markers. Of children who carried *Plasmodium* parasites (36.4% = 123/338), only 1 was infected with *Plasmodium* species other than *P. falciparum*.

**Table 1:** Characteristics of the study population

	All children		Children without inflammation or *Plasmodium* infection	
*n*		338		84
Age				
12–23.99 months	53.6%	(181)	54.8%	(46)
24–36 months	46.4%	(157)	45.2%	(38)
Sex, male	55.0%	(186)	47.6%	(40)
Height-for-age z-score, SD	−1.33	(1.4)	−0.91	(1.5)
Stunted (height-for-age z-score < −2 SD)	30.2%	(102)	14.3%	(12)
Weight-for-height z-score, SD	−0.14	(1.0)	−0.15	(0.97)
Wasted (weight-for-age z-score < −2 SD)	3.0%	(10)	2.4%	(2)
Whole blood ZPP, μmol/mol haem	181	[124–282]	131	[98–239]
Whole blood ZPP > 70 μmol/mol haem	97.9%	(331)	97.6%	(82)
Erythrocyte ZPP, μmol/mol haem	142	[86–246]	117	[69–215]
Erythrocyte ZPP > 70 μmol/mol haem	83.1%	(281)	72.6%	(61)
Erythrocyte ZPP > 40 μmol/mol haem	96.7%	(327)	95.2%	(80)
Haemoglobin concentration, g/L	105.0	(13.2)	111.7	(10.0)
Anaemia (haemoglobin concentration < 108 g/L)	55.6%	(188)	34.5%	(29)
Plasma ferritin concentration, μg/L	35.3	[17.1–67.2]^a^	17.3	[10.3–28.8]^b^
Iron status^c^				
Deficient	17.1%	(57/333)	32.1%	(27/81)
Replete	23.1%	(77/333)	64.3%	(54/81)
Uncertain	59.8%	(199/333)	Not applicable	
Plasma sTfR concentration, mg/L	2.43	[1.84–3.30]	2.11	[1.50–2.94]
Plasma albumin concentration, g/L	34.8	(3.9)	37.4	(2.2)
Plasma vitamin B_12_ concentration, pmol/L	400	[307–559]^d^	356	[283–452]^e^
Plasma vitamin B_12_ concentration < 150 pmol/L	1.2%	(4/332)	1.2%	(1/80)
Plasma CRP concentration, mg/L	2.9	[0.8–9.2]	Not applicable	
Plasma AGP concentration, g/L	1.16	[0.9–1.55]	Not applicable	
Inflammation				
Plasma CRP concentration > 5 mg/L	37.9%	(128)	Not applicable	
Plasma AGP concentration > 1.0 g/L	63.6%	(215)	Not applicable	
Plasma CRP concentration > 5 mg/L, or plasma AGP concentration > 1.0 g/L	66.9%	(226)	Not applicable	
*Plasmodium* infection				
*P. falciparum*	36.1%	(122)	Not applicable	
*Plasmodium* spp. other than *P. falciparum*	0.3%	(1)	Not applicable	
Missing	0.9%	(3)	Not applicable	

Values indicate mean (SD), median [25th and 75th percentile] or % (*n*)

*AGP* *α*_1_-acid glycoprotein, *CRP* C-reactive protein, *sTfR* soluble transferrin receptor, *ZPP* zinc protoporphyrin

Missing values, due to insufficient plasma volumes for analysis, resulted in ^a^ *n* = 333, ^b^ *n* = 81; ^c^ *Deficient:* plasma ferritin concentration < 12 μg/L, regardless of the presence or absence of inflammation; *replete:* plasma ferritin concentration ≥ 12 μg/L, in the absence of inflammation; *uncertain:* plasma ferritin concentration ≥ 12 μg/L, in the presence of inflammation defined as plasma concentrations of CRP > 5 mg/L or AGP > 1.0 g/L. Missing values, due to insufficient plasma volumes for analysis, resulted in ^d^ *n* = 332 and ^e^ *n* = 80

### Factors associated with ZPP: Crude analysis

In bivariate analysis, both whole blood and erythrocyte ZPP were strongly elevated in iron deficiency, anaemia and *Plasmodium* infection; they declined with increasing haemoglobin and plasma ferritin concentrations; and they increased with plasma transferrin receptor concentration (Table 2). Whole blood ZPP was also associated with increased plasma concentrations of α_1_-acid glycoprotein and C-reactive protein concentration; a higher prevalence of inflammation as defined by α_1_-acid glycoprotein >1 g/L; decreased plasma albumin concentration, and increased plasma vitamin B_12_ concentration. Although erythrocyte ZPP was reduced in children aged 24–36 months and, seemed higher in boys than in girls, there was no evidence that it was associated with inflammation, however defined, or with either of the two plasma inflammation markers. We found no evidence that ZPP, whether measured in whole blood or erythrocytes, was associated with z-scores for height-for-age or weight-for-height, or with being stunted or wasted.

**Table 2:** Factors associated with ZPP-haem ratio measured in whole blood or erythrocytes, crude analysis^a^

Factor	Number	Whole blood ZPP-haem ratio			Erythrocyte ZPP-haem ratio		
		Geometric mean	Δ^b^	(95% CI)	Geometric mean	Δ^b^	(95% CI)
Age							
12–23.99 months	162	199	Ref		163	Ref	
24–36 months	176	178	−10.8%	(−21.2% to 0.9%)	134	−18.1%	(−4.3% to −29.9%)
Sex							
Girls	152	179	Ref		135	Ref	
Boys	186	195	9.4%	(−3.4% to 23.8%)	158	16.4%	(−0.5% to 36.1%)
Inflammation (CRP concentration > 5 mg/L)							
No	210	180	Ref		144	Ref	
Yes	128	201	11.4%	(−1.9% to 26.5%)	153	6.6%	(−9.3% to 25.4%)
Inflammation (AGP concentration > 1 g/L)							
No	123	166	Ref		136	Ref	
Yes	215	201	21.4%	(7.0% to 37.9%)	154	13.3%	(−3.7% to 33.3%)
Inflammation (CRP concentration > 5 mg/L or AGP concentration > 1 g/L)							
No	112	166	Ref		137	Ref	
Yes	226	199	20.0%	(5.4% to 36.7%)	153	11.4%	(−5.6% to 31.6%)
Iron deficiency (ferritin concentration < 12 μg/L)							
No	276	173	Ref		132	Ref	
Yes	57	278	60.4%	(36.9% to 87.8%)	255	93.6%	(58.8% to 136.0%)
Anaemia							
No	150	135	Ref		103	Ref	
Yes	188	244	81.4%	(63.3% to 101.4%)	195	89.1%	(64.3% to 117.5%)
*Plasmodium* infection, any species							
No	212	167	Ref		131	Ref	
Yes	123	230	37.5%	(21.5% to 55.6%)	180	38.1%	(18.6% to 60.8%)
Plasma ferritin concentration, 5.11-fold change^d, e^			−16.7%	(−24.6% to −7.9%)		−18.1%	(−24.1% to −11.6%)
Plasma sTfR concentration, 1.43-fold change^d^			38.3%	(34.0% to 42.7.0%)		66.8%	(57.7% to 76.4%)
Plasma CRP concentration, 2.72-fold change^d^			3.5%	(0.8% to 6.2%)		6.8%	(−1.2% to 15.5%)
Plasma AGP concentration, 1.60-fold change^d^			23.7%	(10.0% to 39.1%)		7.1%	(−1.0% to 15.8%)
Haemoglobin concentration, change by 13.2 g/L^f, g^			−29.8%	(−33.1% to −26.2%)		−31.5%	(−35.9% to −26.7%)
Plasma albumin concentration, change by 3.9 g/L^f^			−7.0%	(−12.5% to −1.1%)		−1.4%	(−8.9% to 6.7%)
Plasma vitamin B_12_ concentration, change by 213 pmol/L^f^			7.0%	(0.6% to 13.9%)		6.1%	(−2.0% to 14.8%)

*AGP α*_1_-acid glycoprotein, *CRP* C-reactive protein, *GSD* geometric standard deviation, *Ref* reference, *SD* standard deviation, *sTfR* soluble transferrin receptor, *ZPP* zinc protoporphyrin

^a^ZPP values were normalised by log-transformation; exponentiation of results yielded associations being expressed as relative differences; ^b^Difference; ^c^Based on HRP2- and pLDH-based dipstick test results; ^d^Corresponding to 1 geometric standard deviation; ^e^For example, a 5.11-fold increase in plasma ferritin concentration, which corresponds to a variation that is equivalent to addition of 1 SD on a log-transformed scale, is associated with a reduction in the whole blood ZPP-haem ratio by 16.7%; ^f^Corresponding to 1 SD; ^g^For example, an increase in haemoglobin concentration by 13.2 g/L, which corresponds to an increase by 1 SD, is associated with a reduction in the whole blood ZPP-haem ratio by 29.8%

### Factors associated with ZPP: Multiple linear regression analysis

In multivariate analysis, there was no evidence that ZPP, whether measured in whole blood or erythrocytes, was associated with plasma concentrations of α_1_-acid glycoprotein and vitamin B_12_, or with sex or age class. Thus, these factors were eliminated from the models shown in Table 3.

**Table 3:** Factors associated with ZPP-haem ratio measured in whole blood or erythrocytes, multiple linear regression analysis^a^

	Whole blood ZPP-haem ratio (model 1)		Whole blood ZPP-haem ratio (model 2)		Erythrocyte ZPP-haem ratio	
	Δ^b^	(95% CI)	Δ^b^	(95% CI)	Δ^b^	(95% CI)
Plasma ferritin concentration, 5.11-fold change^c^	−10.1%	(−14.3% to −5.6%)	−9.6%	(−14.0% to −4.9%)	−15.5%	(−21.1% to −9.5%)
Plasma sTfR concentration, 1.43-fold change^c^	34.8%	(28.3% to 41.7%)	34.9%	(28.3% to 41.8%)	45.3%	(35.7% to 55.6%)
Plasma CRP concentration, 2.72-fold change^c^	5.0%	(0.1% to 10.0%)	5.4%	(0.4% to 10.5%)	9.8%	(2.8% to 17.2%)
Haemoglobin concentration, change by 13.2 g/L^d^	−13.9%	(−18.3% to −9.4%)	−14.9%	(−19.4% to −10.1%)	−14.5%	(−20.7% to −7.9%)
*Plasmodium* infection, any species^e^	17.3%	(7.8% to 27.7%)	16.8%	(7.4% to 27.2%)	93.3%	(23.8% to 202.0%)
Plasma albumin concentration, change by 3.9 g/L^d^	―	(eliminated)	3.0%	(−1.7% to 8.0%)	2.2%	(0.5% to 4.0%)

*CRP* C-reactive protein, *sTfR* soluble transferrin receptor, *ZPP* zinc protoporphyrin

^a^ZPP values were normalised by log-transformation; exponentiation of results yielded associations being expressed as relative differences. Plasma concentrations of *α*_1_-acid glycoprotein (log-transformed), vitamin B_12_ (log-transformed), sex (binary) and age class (binary) were eliminated from all models through a manual stepwise backward elimination process with an removal criterion of *p* > 0.05; ^b^Difference; ^c^Corresponding to 1 geometric standard deviation; ^d^Corresponding to 1 SD; ^e^Based on HRP2- and pLDH-based dipstick test results

Whole blood ZPP was independently associated with decreased concentrations of haemoglobin and ferritin and with increased plasma concentrations of transferrin receptor and C-reactive protein (Table 3). Of all biochemical markers, plasma transferrin receptor concentration showed the strongest association with whole blood and erythrocyte ZPP. Similar results were found with erythrocyte ZPP as dependent variable. *Plasmodium* infection was associated with elevated whole blood ZPP, and an even more pronounced elevation in erythrocyte ZPP values. There was a mild association between plasma albumin concentration and erythrocyte ZPP, but retention of this factor in the model for whole blood ZPP did not appreciably change the magnitude of the association for other factors.

### Diagnostic performance of ZPP to detect iron deficiency

**Fig. 1:**
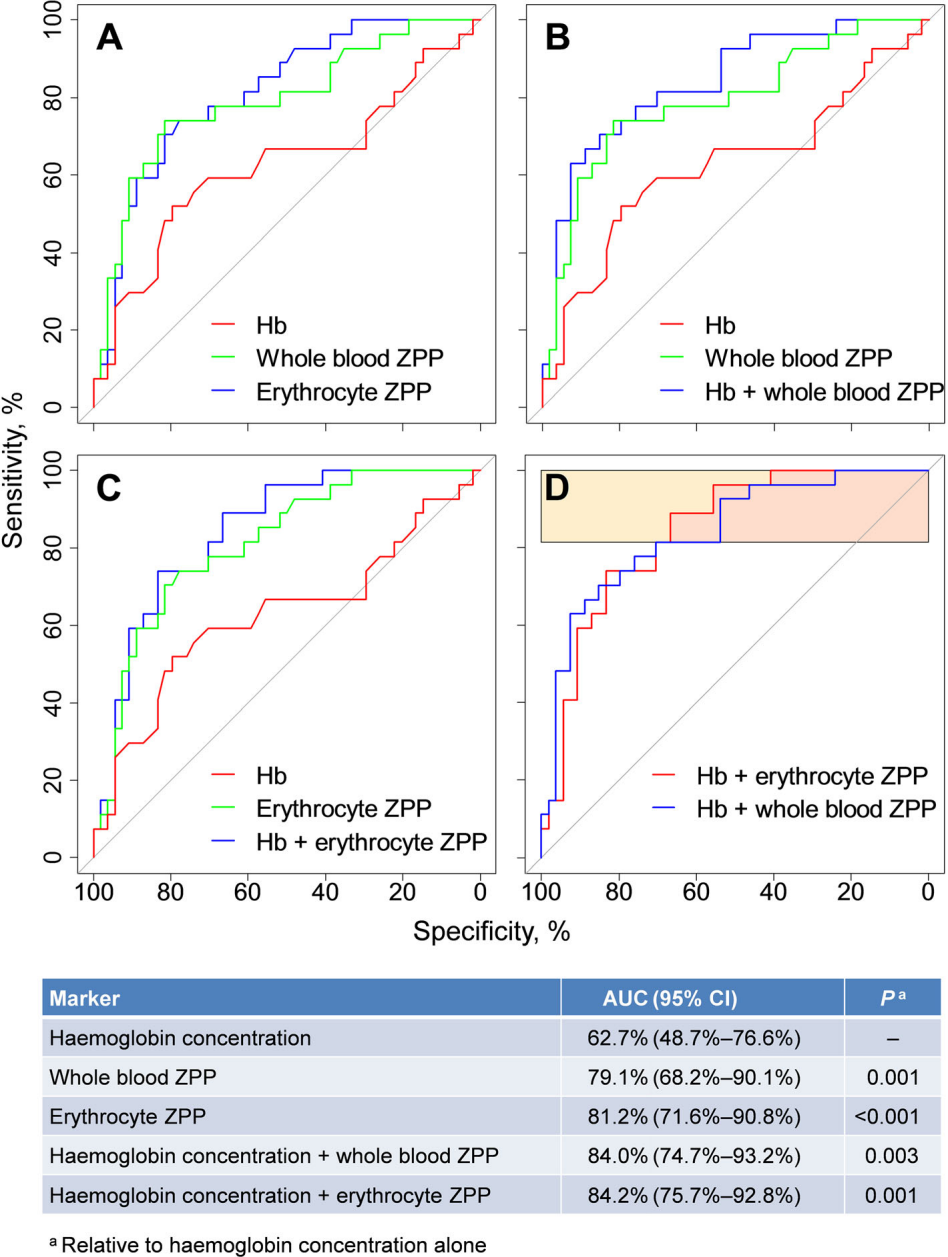
Ability of zinc protoporphyrin-haem ratio, either alone or in combination with haemoglobin concentration, to discriminate between children with and without iron deficiency. Panel **a**: Receiver operating characteristics (ROC) curves for various blood markers, used alone, to discriminate between iron-deficient and iron-replete children. Panel **b**: As in Panel **a**, with haemoglobin concentration and whole blood ZPP, alone and in combination. Panel **c**: As in Panel **a**, with haemoglobin concentration and erythrocyte ZPP, alone and in combination. Panel **d**: As in Panel **a**, with combined haemoglobin concentration and whole blood ZPP, versus combined haemoglobin concentration and erythrocyte ZPP. Hb: haemoglobin concentration. Grey diagonal lines in ROC curves indicate a ‘worst’ possible test, which has no discriminatory value and an area-under-the-curve (AUC) of 0.5. An ideal marker would have a curve that runs from the lower-left via the upper-left to the upper-right corner, yielding an AUC of 1.0

ROC curve analysis showed that whole blood and erythrocyte ZPP had similar diagnostic performance (AUC values: 79.1% and 81.2%, respectively; *p* = 0.36) in detecting iron deficiency, but either marker performed better than haemoglobin concentration (AUC: 62.7%) (Fig. 1a; Table in Fig. 1). The diagnostic accuracy was further improved by combining either whole blood or erythrocyte ZPP with haemoglobin (Fig. 1b, p=0.003; and Fig. 1c, *p*=0.001, respectively).

Overall, there was no evidence that the diagnostic accuracy differed between the combination of haemoglobin concentration with erythrocyte ZPP and the combination of haemoglobin concentration with whole blood ZPP (AUCs: 84.2% versus 84.0%; *p* = 0.91). The ROCs for these markers crossed (Fig. 1d) at a sensitivity of 81.5%, corresponding to 0.07195[Hb] + 0.01449[ZZP_whole_] = 10.39334 (where Hb and ZPP_whole_ indicate haemoglobin concentration in g/L and whole blood ZPP:haem ratio in μmol/mol, respectively; in this equation, parameter estimates are shown with 5 decimals to avoid misclassification due to multiplication of rounding errors). At all sensitivity values above this cut-off (Fig. 1d, red rectangle), the diagnostic accuracy of the combination of erythrocyte ZPP with haemoglobin concentration was superior to the combination of whole blood ZPP with haemoglobin concentration (corrected pAUCs: 76.3% versus 70.4%, *p* = 0.04).

### Diagnostic utility of ZPP

When whole blood ZPP was considered without additional markers to detect iron deficiency, a conventional threshold of 70 μmol/mol haem resulted in the following estimates **(**Table 4 **)**: sensitivity: 100%; specificity: 3.7%, positive predictive value: 34.2%; prevalence: 97.5% (as compared to a ‘true’ prevalence of 32.1%; Table 1). Corresponding values for an erythrocyte ZPP threshold of 40 μmol/mol haem were: 100%, 7.4%, 35.1% and 95.1%.

**Table 4:** Diagnostic performance of zinc protoporphyrin-haem ratio, with dichotomised test results, to detect iron deficiency

	n/n	Estimate	(CI)
Whole blood ZPP > 70 μmol/mol haem			
Sensitivity	27/27	100.0%	(84.4%–100.0%)^a^
Specificity	2/54	3.7%	(0.9%–14.4%)^a^
Positive predictive value	27/79	34.2%	(23.5%–46.7%)^a^
Negative predictive value	2/2	100.0%	(28.6%–100.0%)^a^
Prevalence	79/81	97.5%	(91.4%–99.3%)^b^
Erythrocyte ZPP > 40 μmol/mol haem			
Sensitivity	27/27	100.0%	(84.4%–100.0%)^a^
Specificity	4/50	7.4%	(2.6%–19.5%)^a^
Positive predictive value	27/77	35.1%	(24.2%–47.8%)^a^
Negative predictive value	4/4	100.0%	(44.4%–100.0%)^a^
Prevalence	77/81	95.1%	(88.0%–98.1%)^b^

^a^97.5% CI; ^b^95% CI

**Fig. 2:**
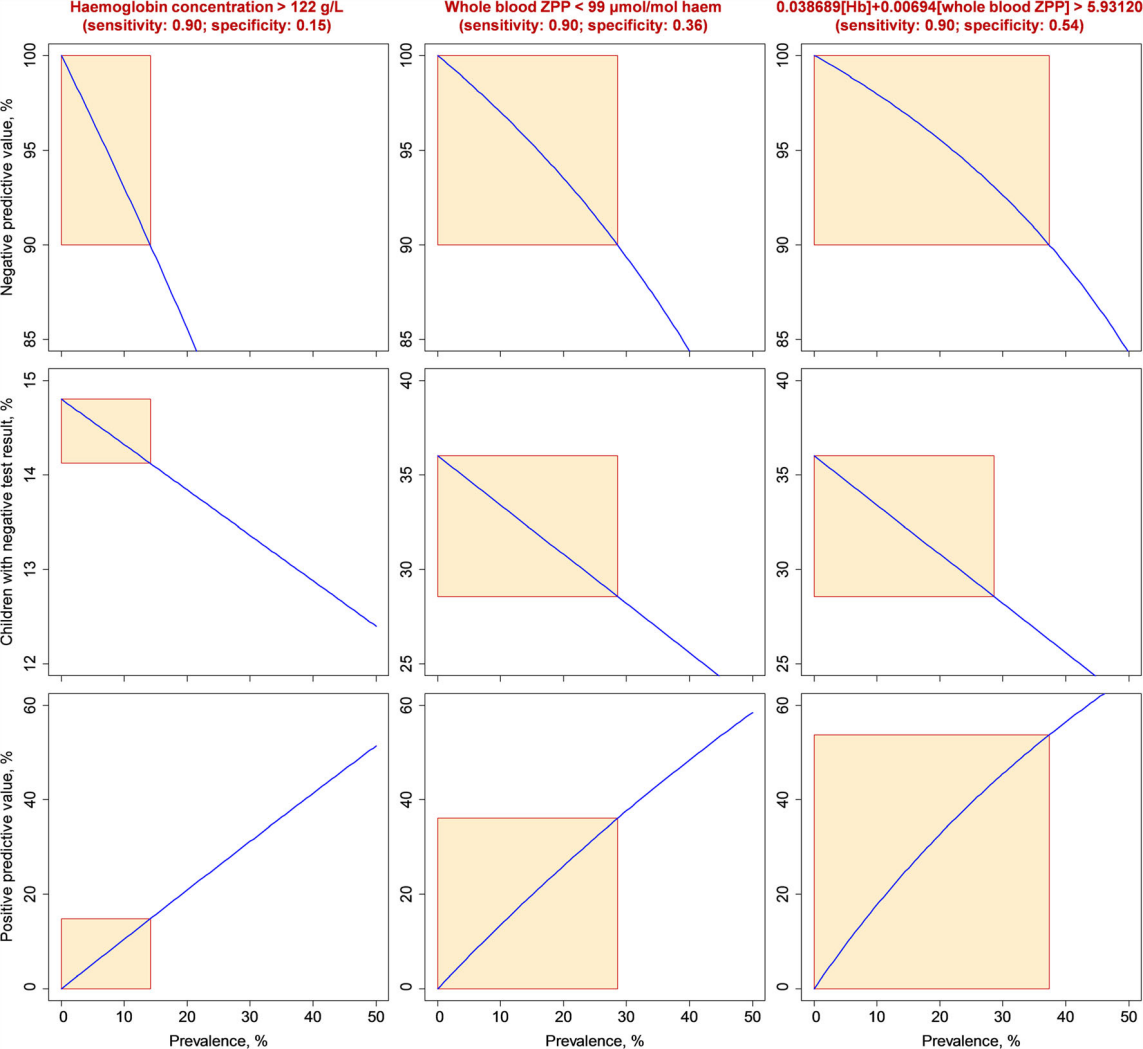
Application of a diagnostic strategy to rule out iron deficiency. [Hb]: haemoglobin concentration, expressed in g/L; [whole blood ZPP]: whole blood ZPP content, expressed in μmol/mol haem. The diagnostic strategy in a screen-and-treat survey is based on two criteria: a) the probability of correctly diagnosing iron deficiency should exceed 90%; and b) iron deficiency can be ruled out if the probability of a negative test result being correct (negative predictive value) exceeds 90%. To meet the first requirement, a cut-off point for each diagnostic test result (top) is selected to yield a sensitivity of 90%; the corresponding specificity value is obtained from the ROC curves (Fig. 1). For haemoglobin concentration and whole blood ZPP, the cut-off points were 122 g/L and 99 μmol/mol haem; the corresponding specificity values were 14.8% and 36.0%, respectively. When these markers were combined in a single diagnostic rule, 0.038689 [Hb] + 0.00694 [whole blood ZPP] > 5.93120 had a specificity of 53.7%. The negative predictive value (top panels, *blue* lines) depends on sensitivity and specificity values thus fixed, and the prevalence of iron deficiency. The second diagnostic criterion, i.e. the negative predictive value should exceed 90%, applies only within a limited prevalence range (top panels, red rectangle); at prevalence values exceeding this range, the negative predictive value will be below 90% and iron deficiency cannot be ruled out with diagnostic test applied (top). The percentage of children with a negative test result declines linearly with the prevalence of iron deficiency (middle panels, blue lines). The percentage of children for whom iron deficiency can be ruled out (middle panels, Y-intercepts of red rectangles) depends on the prevalence range in which the negative predictive value exceeds 90% (top panels, red rectangles). With fixed sensitivity and specificity values, the positive predictive value (bottom panels, blue lines) increase monotonically with the prevalence of iron deficiency. Within the prevalence range in which the negative predictive value exceeds 90% (top panels, *red* rectangles), the highest positive predictive value is 54% (for combined use of haemoglobin concentration and whole blood ZPP), indicating that additional tests (i.e. other than haemoglobin concentration and whole blood ZPP) are required to accurately determine iron status. For example, haemoglobin concentration > 122 g/L (left panels) has 90% sensitivity of detecting iron deficiency; at a true prevalence of iron deficiency <14.1%, a negative test result obtained with this decision rules out iron deficiency with a probability of 90% (upper left panel, red rectangle). Depending on the true prevalence of iron deficiency, such a cut-off for haemoglobin concentration would result in iron deficiency being ruled out in 14.1%–14.8% of children who are tested (middle left panel, red rectangle). Similarly, within a prevalence range < 28.6%, whole blood ZPP > 99 μmol/mol haem rules out iron deficiency in 28.6%–36% of children tested. Within a prevalence range of <37.4%, 0.038689 [Hb] + 0.00694 [whole blood ZPP] > 5.93120 rules out iron deficiency in 37.4%–53.7% of children

Within a prevalence range of iron deficiency of <14.1%, a diagnostic rule of haemoglobin concentration > 122 g/L would rule out iron deficiency in 14.1%–14.8% of children tested, depending on the actual prevalence, with both sensitivity and negative predictive value >90% (Fig. 2). Similarly, within a prevalence range of iron deficiency of <28.6%, whole blood ZPP > 99 μmol/mol haem would rule out iron deficiency in 28.6%–36% of children tested; and within a prevalence range of 37.4%, 0.038689 [Hb] + 0.00694 [whole blood ZPP] > 5.93120 would rule out iron deficiency in 37.4%–53.7% of children. At all prevalence values exceeding these ranges, these diagnostic tests would not be able to rule out children with the predefined diagnostic criteria (i.e. both sensitivity and negative predictive value should be 90%), and all children would need to undergo further diagnostic work-up using more advanced tests.

## Discussion

In our population, virtually all children had whole blood ZPP values exceeding conventional cut-off points of 70 μmol/mol haem, resulting in very low specificity and gross overestimates of the ‘true’ prevalence of iron deficiency of 32.1%, whether assessed in the overall population or when restricted to those without inflammation and without *Plasmodium* infection. A similar problem was noted with erythrocyte ZPP > 40 μmol/mol haem. Both whole blood ZPP and erythrocyte ZPP were independently associated with *Plasmodium* infection and plasma C-reactive protein concentration. ZPP, whether measured in whole blood or erythrocytes, yielded higher diagnostic accuracy in detecting iron deficiency than haemoglobin concentration alone, and also improved this diagnostic accuracy when used in combination with haemoglobin concentration. When applied in a screen-and-treat strategy to control iron deficiency in paediatric populations with a prevalence of iron deficiency of <37.4% (which covers most settings in developing countries), our data suggest that a diagnostic rule of 0.038689 [Hb] + 0.00694 [whole blood ZPP] > 5.93120 can correctly identify 90% of children with iron deficiency, and correctly rule out iron deficiency in 37.4%–53.7% of children who are tested, depending on the true prevalence.

Lead poisoning can cause elevated ZPP values, but lead exposure is presumably low in rural African children. Thus, the high ZPP values found in this study population may have been due to a combination of factors causing an inadequate supply of iron to erythroblasts (iron deficiency, inflammation) and increased erythropoiesis (haemolysis due to *Plasmodium* infection and possibly selected hereditary disorders such as glucose-6-phosphate dehydrogenase deficiency, sickle cell and α^+^-thalassaemia). In a previous trial in the same area as the present study, α^+^-thalassaemia occurred in 48.8% of pregnant women (heterozygotes: 41.3%; homozygotes: 7.5%), but there was no evidence that it was associated with ZPP [4]. In a nearby area, the prevalence of sickle cell trait and sickle cell disease in preschool children was 17.1% and 1.6%, respectively; genotypes indicating G6PD deficiency occurred in 8.2% of males and 6.8% of children overall, whilst the prevalence of haptoglobin 2–2 genotype was 20.4% [17, 18] A study in Gambian children aged 2–6 years, however, failed to find an association between haptoglobin genotype and ZPP [19].

We found a particularly strong relationship between ZPP, whether measured in whole blood or erythrocytes, and plasma transferrin receptor concentration. This is not surprising because both are markers for iron-deficient erythropoiesis. Consistent with our data, ZPP is known to be increased in iron deficiency, inflammation and other causes of an inadequate iron supply to erythroblasts. The increase in whole blood ZPP that was associated with *Plasmodium* infection may be due in part to the formation of bilirubin and other haemoglobin breakdown products in plasma that result from haemolysis and that fluoresce in the same wavelength range as protoporphyrins. *Plasmodium* infection was also associated with an even larger increase in erythrocyte ZPP, independently of inflammation. This was unexpected because erythrocytes lack plasma constituents, which are removed by washing. A possible explanation is that haemolysis-induced increase in erythropoietin activity under influence of *Plasmodium* infection drives up the demand for iron in the erythron. We have not been able to find previous reports of an association between erythrocyte ZPP and plasma albumin concentration, which could be a spurious finding.

One limitation of our study is the difficulty of measuring iron status in the presence of inflammation. Throughout the remainder of this discussion, it should be noted that we assessed the diagnostic performance and ZPP in children without inflammation and without *Plasmodium* infection, because plasma ferritin concentration can be elevated in the presence of infection-induced inflammation independently of iron status. We used this approach in favour of other biomarkers and approaches that have been proposed.

In one method [20, 21], fixed correction factors for serum ferritin concentrations are computed based on geometric mean values in groups that are defined by cross-classification of individuals by two inflammation markers (serum concentrations of C-reactive protein >5 g/L and α_1_-acid glycoprotein >1.0 g/L). The resulting ratios in geometric mean values are used to adjust individual values, and deficiency is determined on the basis of these adjusted values. This method has the disadvantages, however, that it is not validated using a reference standard, its validity depends on untested and perhaps invalid assumptions, and it does not take into account likely between-person variability in the response of ferritin concentration to inflammation. Another limitation is the possibility that plasma ferritin concentration is possibly elevated at levels of inflammatory markers within the normal range (serum C-reactive protein concentration < 5 mg/L or plasma *α*_1_-acid glycoprotein concentration < 1.0 g/L). This limitation also applies, of course, to the restriction method used in the analysis of the present paper.

Recently, it has been proposed to adjust values using regression analysis [12]. Although this approach offers several theoretical advantages, its validity remains to be investigated by comparison with a reference standard. The ratio of concentrations of soluble transferrin receptor/log ferritin has been suggested as a marker of body iron content but its use to detect iron deficiency remains problematic for reasons discussed elsewhere [4].

Our results clearly show that, in the absence of inflammation or *Plasmodium* infection, ZPP has added diagnostic value in detecting iron deficiency over haemoglobin concentration alone. This added value applied both to ZPP measured in whole blood or erythrocytes. By contrast, we found that the diagnostic performance of haemoglobin concentration in detecting iron deficiency was similar in pregnant women (AUC: 61%, [4]) as in children (AUC: 63%, present study); in pregnant women, however, replacement of haemoglobin concentration by ZPP, or addition by ZPP to haemoglobin concentration, whether measured in whole blood or in erythrocytes, had little diagnostic value [4].

When combined with haemoglobin concentration, whole blood ZPP or erythrocyte ZPP yielded AUC values for the ROC curves of approximately 84%. Whether this accuracy is satisfactory depends on the purpose of testing. Conventional cut-off points for whole blood ZPP and erythrocyte ZPP are clearly inappropriate and produce gross overestimates of prevalence and a very low positive predictive value (Table 5 in this paper; see also [4]) due to their low specificity. Mwangi et al. 2014 [4], reported how cut-off points can be manipulated to minimize bias in the estimation of population prevalence. In the present paper, we have shown how cut-off points for haemoglobin and whole blood ZPP, either alone or in combination, can be calibrated in a screen-and-treat strategy to identify individuals with iron deficiency at community level. Screening generally requires the selection of a cut-off point to ensure a high sensitivity for the test or combination of tests to be employed. Such high sensitivity ensures that most cases are detected, at the cost of false positives that can be eliminated by further diagnostic tests. Although the accuracy of this approach is insufficient to give a final diagnosis in all individuals, the combination of haemoglobin concentration and whole blood ZPP can constitute a rapid and convenient method to rule out iron deficiency in a substantial proportion of children screened. Although we considered the example of screening at community level, a similar strategy can be employed in clinical practice to identify children who may need referral to higher levels of care for further testing. When 0.07195[Hb] + 0.01449[ZZP_whole_] < 10.39334, the diagnostic accuracy may be improved by using a combination of erythrocyte ZPP with haemoglobin concentration, but the improved diagnostic accuracy thus achieved must be weighed against the procedure of washing red cells, which may be cumbersome in practice.

One problem that has not been solved in our study is the difficulty in detecting iron deficiency in the presence of inflammation. The associations found between ZPP and *Plasmodium* infection and between ZPP and C-reactive protein, underscore the importance of this issue. Further research is required to extend and validate our approach with appropriately selected reference standard for iron deficiency in a population that included individuals with inflammation and infections. Because point-of-care tests are rapidly developing, with quantitative tests already being commercially available for plasma concentrations of ferritin and C-reactive protein, and for various infections, this is likely to be a fruitful area of future research.

## Conclusion

In Kenyan preschool children, ZPP, whether measured in whole blood or in erythrocytes, has added diagnostic value in detecting iron deficiency over haemoglobin concentration alone. When used in a screen-and-treat approach, combination of haemoglobin concentration and whole blood ZPP in a single diagnostic score can be used as a rapid and convenient testing method to rule out iron deficiency in a substantial proportion of children screened.

## Additional file

Additional file 1: The utility of haemoglobin concentration and ZPP as diagnostic tests to screen and rule out iron deficiency. (DOCX 21 kb)

## Abbreviations

AGP: α_1_-acid glycoprotein
AUC: Area-under-the-curve
CRP: C-reactive protein
EP: Erythrocyte protoporphyrin
FEP: Free erythrocyte protoporphyrin
Hb: haemoglobin concentration
HRP2: Histidine-rich protein-2
IPT: Intermittent preventive treatment
pLDH: *Plasmodium* lactate dehydrogenase
ROC: Receiver operating characteristics
WHO: World Health Organization
ZPP: Zinc protoporphyrin
