# Supplementary material for: Diagnostic utility of zinc protoporphyrin to detect iron deficiency in Kenyan preschool children: a community-based survey
Source: BMC Hematol. 2017 Jul 27;17:11. doi: 10.1186/s12878-017-0082-z (PMC5532779; doi:10.1186/s12878-017-0082-z)
Supplement: Additional file 1: — The utility of haemoglobin concentration and ZPP as diagnostic tests to screen and rule out iron deficiency. (DOCX 21 kb) [file 12878_2017_82_MOESM1_ESM.docx]

### Additional file 1: The utility of haemoglobin concentration and ZPP as diagnostic tests to screen and rule out iron deficiency

Consider the example of a community survey in which children are screened for iron deficiency, with treatment being restricted to those who are iron deficient.

In the absence of inflammation, serum ferritin concentration < 12 μg/L is recommended by WHO to measure iron status in paediatric populations [1]. Although commercial point-of-care tests for serum ferritin concentration have recently become available, its measurement is often difficult under field conditions and relatively expensive. A screening strategy whereby haemoglobin concentration and whole blood ZPP are used to rule out iron deficiency would be useful and could possibly lead to substantial cost savings.

Let the result of a diagnostic test (i.e. haemoglobin concentration and ZZP, used alone or in combination) be dichotomized. Table 1 shows the cross-tabulated data that might be obtained.

**Table 1. Cross-tabulated data for true iron status and diagnostic test results**

| Diagnostic test result | Iron deficient (reference) | | Total |
| --- | --- | --- | --- |
|  | Yes | No |  |
| Positive | A | B | U |
| Negative | C | D | V |
| Total | R | T | N |
| Capital letters in each cell indicate the number of individuals in that cell | | | |

The aim of screening is to detect most cases of iron deficiency, i.e. with a cut-point of the test result selected to ensure a high sensitivity (e.g. >90%). Because a high sensitivity comes at the cost of a reduced specificity, this will inevitably result in false positives that could be eliminated by further diagnostic tests. The advantage of screening with a high sensitivity, however, is that iron deficiency can be effectively and with near-certainty be ruled out in those with a negative test result [2].

Let the negative predictive value (*NPV* = D/V) be set to ensure that a high proportion of negative test results (e.g. >90%) are correct. The *NPV* can be rewritten as a function of the prevalence, sensitivity and specificity:

$$NPV=\frac{D}{V}=\frac{T\cdot Spec}{C+D}=\frac{\left( N-R \right)\cdot Spec}{R\cdot\left( 1-Sens \right)+\left( N-R \right)\cdot Spec}$$

Dividing both numerator and enumerator by *N* yields:

| $NPV=\frac{\left( 1-P \right)\cdot Spec}{P\cdot\left( 1-Sens \right)+\left( 1-P \right)Spec}$ |
| --- |

Thus, at a sensitivity of 90% and corresponding specificity derived from the ROC curves (Figure 1), a *NPV* > 90% can be obtained only within a certain prevalence range.

Similarly, the positive predictive value (*NPV* = A/U) can be formulated as a function of the prevalence, sensitivity and specificity:

| $PPV=\frac{P\cdot Sens}{P\cdot Sens+\left( 1-P \right)\cdot\left( 1-Spec \right)}$ |
| --- |

The fraction with a negative test result (*F* = V/N) for whom iron deficiency can be ruled out can similarly be rewritten as a function of the prevalence or pre-test probability (*P* = R/T), sensitivity (*Sens* = A/R) and specificity (*Spec* = D/T) as follows:

$$F=\frac{V}{N}=\frac{\left( C+D \right)}{N}=\frac{R\cdot\left( 1-Sens \right)+T\cdot Spec}{N}=\frac{R\cdot\left( 1-Sens \right)}{N}+\frac{(N-R)\cdot Spec}{N}$$

Thus:

| $F=P\cdot\left( 1-Sens \right)+\left( 1-P \right)\cdot Spec=Spec+P\cdot\left( 1-Sens-Spec \right)$ |
| --- |

As indicated by this formula, when the sensitivity is arbitrarily set at >90%, and the corresponding specificity is derived from the ROC curves (Figure 1), *F* varies only with the prevalence of iron deficiency.

In summary, by screening with a cut-point for the diagnostic test result that is preselected to yield a high sensitivity (e.g. > 90% or > 95%), one can determine a prevalence range in which the negative predictive value that is within an acceptably high range (e.g. > 90% or > 95%) and that produces a variable fraction of persons in whom iron deficiency can be ruled out (Figure 2).

Reference

1. WHO/CDC: *Assessing the iron status of populations, 2nd ed.* Report of a joint World Health Organization/Centers for Disease Control and Prevention Technical Consultation on the assessment of iron status at the population level (Geneva, Switzerland: 6–8 April 2004). Geneva, Switzerland: World Health Organization; 2007
2. Thurnham DI, McCabe LD, Haldar S, Wieringa FT, Northrop-Clewes CA, McCabe GP: Adjusting plasma ferritin concentrations to remove the effects of subclinical inflammation in the assessment of iron deficiency: a meta-analysis**.** *Am J Clin Nutr* 2010; **92:**546-55
